# Intra-Session Reliability and Predictive Value of Maximum Voluntary Isometric Contraction for Estimating One-Repetition Maximum in Older Women: A Randomised Split-Sample Study

**DOI:** 10.3390/jfmk10020160

**Published:** 2025-05-06

**Authors:** José Aldo Hernández-Murúa, Ena Monserrat Romero-Pérez, Jorge Luis Guajardo-Cruztitla, Blas Sinahí Madrigal Olivares, Ángel Gallego-Selles, Diego González-Martín, Francisca Reyes-Merino, Nidia Sánchez-García, José Antonio de Paz

**Affiliations:** 1 Faculty of Physical Education and Sports, Autonomous University of Sinaloa (UAS), Culiacán 80013, Mexico; aldohdez80@hotmail.com (J.A.H.-M.); jorge.guajardo@uas.edu.mx (J.L.G.-C.); sinahimadrigal0905@gmail.com (B.S.M.O.); 2 Division of Biological Sciences and Health, University of Sonora, Hermosillo 83000, Mexico; 3 Department of Physical Education and, Research Institute of Biomedical and Health Sciences (IUIBS), University of Las Palmas Gran Canaria, 35017 Las Palmas Gran Canaria, Spain; angelgallegoselles@hotmail.com; 4 Institute of Biomedicine (IBIOMED), University of León, 24071 León, Spain; diegogmgi3h@gmail.com (D.G.-M.); freyem00@estudiantes.unileon.es (F.R.-M.); nisag@unileon.es (N.S.-G.); japazf@unileon.es (J.A.d.P.)

**Keywords:** muscle strength assessment, aging, predictive validity, leg extension

## Abstract

**Background:** Ageing is associated with a progressive decline in muscle strength, particularly in the lower limbs, which compromises functional independence. While both maximum voluntary isometric contraction (MVIC) and one-repetition maximum (1RM) are widely employed to assess muscle strength, the intra-session reliability and predictive capacity of MVIC for estimating 1RM in older women remain insufficiently explored. **Objectives:** This study aims to evaluate the intra-session reliability of MVIC in knee extensors, analyse its correlation with 1RM, and develop a predictive model for estimating 1RM from MVIC in older women. **Methods:** Using a randomised split-sample design, 82 women aged 60–69 years performed two MVIC trials and one 1RM test using a leg extension machine. Intra-session reliability was assessed by calculating the intraclass correlation coefficient (ICC), the standard error of measurement (SEM), and the minimal detectable change (MDC). Furthermore, a linear regression model was developed to predict 1RM based on MVIC. **Results:** MVIC demonstrated excellent intra-session reliability (ICC = 0.96, SEM = 4.3%, MDC = 11.9%), and a strong correlation between MVIC and 1RM was observed (R^2^ = 0.618). Although the predictive equation 1RM = [(0.932 × MVIC) − 3.852] did not yield statistically significant differences between the estimated and actual 1RM values (*p* = 0.791), it exhibited a prediction error of 13.4%. **Conclusions**: MVIC is a highly reliable measure in older women and represents a practical tool for estimating 1RM. Nonetheless, its predictive accuracy is limited, highlighting the need for further studies to refine predictive models by incorporating additional variables.

## 1. Introduction

Muscle strength is defined as the ability of the musculoskeletal system to generate tension through the contraction of sarcomeres within muscle cells. The magnitude of the generated tension and the nature of the muscle action determine the various manifestations of strength [1]. It is estimated that, from the age of 50 to 60, older adults experience a progressive decline in both muscle strength (approximately 1.5–5% per year) [2] and muscle mass (around 1% per year) [3]. This decline is more pronounced in the lower limbs than in the upper limbs, particularly following periods of physical inactivity [4]. Strength training has been shown to be effective in mitigating these losses and in providing additional health benefits [5]. It promotes the preservation of functional independence and mobility, reduces the risk of falls, frailty, sarcopenia, and fractures, and lowers all-cause mortality [6]. In fact, the World Health Organisation and the American Heart Association recommend that strength training be individually prescribed, considering an individual’s maximum strength and applying progressive, systematic increases to optimise both muscular and cardiovascular health [5,7].

Two widely used methods for assessing muscle strength are the one-repetition maximum (1RM), which measures the maximum load an individual can lift with proper technique, and maximum voluntary isometric contraction (MVIC). The 1RM is widely regarded as the gold standard for both programming and quantifying exercises involving dynamic contractions. However, its determination typically requires at least two sessions, one for familiarisation and another for evaluation. Furthermore, the 1RM assessment requires specific equipment (e.g., dumbbells, weighted elements, or resistance machines), which may limit its applicability in certain settings [8]. In clinical practice, patient conditions can also impede the accurate assessment of 1RM [9]. Consequently, some trainers and researchers choose to estimate 1RM based on the statistical relationship between a given load, the maximum number of repetitions performed with that load, and the corresponding 1RM [10]. In addition, emerging methods propose estimating 1RM from the relationship between the load and the speed at which it is lifted, offering an alternative approach that may be less stressful [11]. Given these limitations associated with 1RM assessments, alternative methods have been sought to provide safer and more practical means of evaluating muscle strength.

In contrast, MVIC represents a simpler and more practical alternative for assessing maximum strength, as it requires only a mechanical dynamometer or load cell. This method quantifies the tension generated by a muscle contraction against an unyielding resistance and is widely used in both rehabilitation and research settings for functional assessment and exercise prescription. Moreover, it has been successfully applied in healthy older adults, as well as in patients with chronic degenerative conditions, such as hypertension, osteoarticular disorders, or neuromuscular disorders [12,13,14].

In this context, several studies have utilised MVIC to estimate training loads and examine their effects in dynamic exercises [15,16]. In addition, research among young adults has demonstrated a high correlation between MVIC and 1RM, suggesting that these measures can serve as complementary indicators of muscle strength [17].

Given that both MVIC and 1RM are fundamental for individualising training loads and evaluating training outcomes, it is essential to ascertain the intra-session reliability of these measurement procedures. Repeatability studies, which establish the standard error of the method (SEM) and the minimum detectable change (MDC), provide valuable insights into interpreting result magnitudes and distinguishing between genuine intervention-induced changes and random measurement variations.

Studies on the intra-session reliability of MVIC in knee extensors have reported internal consistency values ranging from good to excellent in both adults and older adults (ICC ≥ 0.9, CV ≤ 5%). Notably, whereas assessments in young adults have predominantly employed the isometric mid-thigh pull, evaluations in older adults more commonly utilise the knee extension test (leg extension) [18,19,20]. Despite the existing evidence supporting the relationship between 1RM and MVIC, studies exploring this association in bilateral knee extension exercises among older adults remain scarce [17,21,22]. Moreover, research specifically focused on older women is limited, thereby representing a significant gap in current knowledge and emphasising the need for targeted studies in this population.

The objectives of this study were to assess the intra-session reliability of maximum voluntary isometric contraction (MVIC) measurement in the knee extensors among older women, to analyse the relationship between MVIC and one-repetition maximum (1RM), and to determine the predictive value of MVIC for estimating 1RM.

## 2. Materials and Methods

### 2.1. Study Design

A randomised split-sample design was employed, whereby all participants underwent two MVIC measurements and one 1RM measurement of the knee extensor muscles. Furthermore, a cross-validation design was implemented to analyse the predictive value of MVIC. For this purpose, participants were sequentially numbered according to the order of their assessment to facilitate random assignment to either the study group (SG) or the validation group (VG). Subsequently, 41 random numbers were generated using the online application “https://echaloasuerte.com/number” (accesed on 15 January 2025). Participants whose assigned numbers were among the 41 randomly generated were allocated to the SG, while the remaining participants constituted the VG.

### 2.2. Participants

To recruit participants, informational sessions about the project were held at senior civic centres in the municipality of Culiacán, where educational and cultural activities were provided, although no physical exercise programmes were offered. Interested individuals were scheduled for an interview at the Exercise Laboratory of the Faculty of Physical Education and Sport of the Autonomous University of Sinaloa, (Culiacán, Mexico). A total of 82 older women were recruited, and after being informed about the objectives of the study, they provided written informed consent for voluntary participation. Data collection took place between November 2022 and January 2023. The research protocol was approved by the Research Ethics Committee of the Universidad Autónoma de Sinaloa (7 December 2021, protocol ID: PRO_A3_047) and was conducted in accordance with ethical procedures, international standards, and the Declaration of Helsinki. Before testing, participants underwent a medical interview and physical examination to confirm that they had no medical contraindications for performing maximum muscle strength assessments.

The inclusion criteria required participants to be 60 years or older, not institutionalised, and have no prior experience in strength training. Participants were excluded if they had any contraindication that affected their ability to understand instructions or safely perform muscle strength tests.

### 2.3. Determination of Maximum Voluntary Isometric Contraction (MVIC)

**Figure 1:**
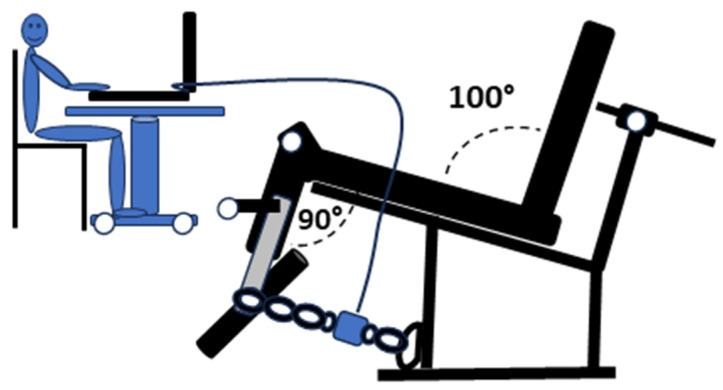
Placement of the load cell for the assessment of maximum voluntary isometric contraction (MVIC) during the knee extension leg exercise.

The MVIC and 1RM assessments for the knee extensors were performed using a Scom^®^ Line knee extension machine (Professionals Body Building and Fitness^®^, Tlaquepaque, México), with a 100° angle between the seat and backrest. The maximum voluntary isometric contraction (MVIC) of the knee extensors was measured using a load cell connected to its corresponding software (Chronojump Bosco System^®^, version 2.3.0-1, Barcelona, Spain). The testing procedure followed the methodology described in previous studies [16,23]. The load cell was positioned between two chain segments equipped with carabiners, with one end attached to the central support of the machine and the other secured to the push lever (Figure 1). Before the test, participants completed a 5 min warm-up on a cycle ergometer, pedalling at 60 rpm with an approximate load of 25 W. The knee flexion angle was adjusted between 90° and 95°, depending on leg length, using a goniometer (GIMA, Model 27340, Gessate, Italy). This adjustment was made by modifying the number of chain links, while the seat-backrest angle was set at 100°.

For the MVIC trial, participants were instructed to exert maximum force explosively upon the start signal and maintain it for 5 s [16,23]. Throughout the effort, strong verbal encouragement was provided to help sustain maximal force output. Two trials were performed, with a 5-min recovery interval between them. For intra-session reliability analysis, both MVIC trials were considered, whereas, for the linear regression analysis, the highest value recorded from the two attempts was used.

### 2.4. One-Repetition Maximum (1RM) Assessment

The 1RM was determined using a standardised testing protocol, conducted ten minutes after the final MVIC measurement. Before the test, participants performed a specific warm-up consisting of two sets of six repetitions at 50% of MVIC, with a three-minute rest between sets. After a further three-minute recovery, successive sets of two repetitions were initiated. At the end of each set, participants rated their perceived effort using the OMNI-RES strength scale.

The test began with an initial load of 15 kg above the weight used in the warm-up sets. If a set was successfully completed, a subsequent set was initiated two minutes later, with the load increased by 2 to 10 kg, depending on the perceived difficulty and the quality of technical execution. If proper technique was not maintained, the weight was reduced to an intermediate value between the last correctly completed set and the failed attempt.

This process was repeated until the maximum load that each participant could lift once with proper technique—defined as completing at least approximately 75% of the knee extension range of motion achieved with the initial load—was determined and recorded as their 1RM. A maximum limit of six sets was established to reach the 1RM [24].

### 2.5. Statistical Analysis

Descriptive results are presented as mean ± standard deviation (SD). Normality was assessed using the Kolmogorov–Smirnov test. The reliability of MVIC was analysed using the intraclass correlation coefficient (ICC) with a two-factor random-effects model and absolute agreement for single measurements (ICC_2,1_). Additional reliability metrics included the coefficient of variation (CV), the standard error of measurement (SEM) (SEM = SD × √(1 − ICC)), and the minimal detectable change (MDC) (MDC_95_ = SEM × 1.96 × √2).

To examine the relationship between MVIC and 1RM, a linear regression analysis was conducted in the study group (SG), and the resulting equation was validated in the validation group (VG) by comparing the estimated 1RM with the measured 1RM. A paired *t*-test was used to compare measured and estimated 1RM values, while an independent *t*-test was applied to compare absolute percentage errors between the SG and VG. The absolute percentage error between measured and estimated 1RM was calculated using the following equation: Absolute Percentage Error (%) = √[($\frac{estimated\;1RM\;\times \;100}{measured\;1RM}$) − 100].

The a priori sample size calculation was performed using G*Power (version 3.1.9.7, Heinrich Heine University, Düsseldorf, Germany) according to the following procedures:

(a) To estimate the correlation between MVIC and 1RM, an exact test for a two-tailed bivariate correlation was selected, with an effect size (ρ) of 0.5, α = 0.05, and 1-β = 0.8, yielding a minimum sample size of 46 participants.
(b) For the intra-session reliability and MDC determination, a two-tailed paired *t*-test was chosen, with Cohen’s d = 0.5, α = 0.05, and 1-β = 0.80, resulting in a minimum sample size of 34 participants.
(c) To assess the predictive value of MVIC for 1RM, an F-test for multiple linear regression (fixed model, R^2^ increase) was selected, with an effect size (f^2^) of 1.77 (80% variance explained), a single predictor (MVIC), α = 0.05, and 1-β = 0.80, yielding a minimum sample size of 47 participants. All statistical analyses were performed using SPSS 25.0 (IBM Inc., Chicago, IL, USA).

## 3. Results

Table 1 presents the physical characteristics of the sample, separated into the study group (*n* = 41) and the validation group (*n* = 41). No significant differences were observed between the groups for any of the analysed variables.

Table 2 shows the reliability analysis results for MVIC measurements, demonstrating excellent intra-session reliability, with an ICC of 0.960, an SEM of 4.3%, and an MDC of 11.9%.

Table 3 displays the linear regression parameters obtained for the study group, including the regression equation (R^2^ = 0.618, SEE = 6.9 kg), indicating that the model demonstrates an acceptable predictive capacity within the study group.

Table 4 provides a comparison between the measured 1RM and the estimated 1RM using the study group’s regression equation for both groups. No significant differences were observed between the estimated and measured values in either group. Additionally, the table presents the percentage differences between the measured and estimated 1RM within each group, and the comparison of these differences between groups was not statistically significant.

## 4. Discussion

The present study assessed the intra-session reliability of maximum isometric strength in knee extensors among older women, as well as its predictive capacity for estimating dynamic maximum strength. The sample consisted exclusively of women with an average age of 67 years. It is important to emphasise that older adults are not a homogeneous group in terms of functionality, health, or needs.

Defining ageing solely on the basis of a dichotomous chronological criterion (i.e., older adult vs. non-older adult based on an age cutoff) is limiting. Ageing is a gradual process spanning approximately 20 years, during which physical capacities progressively decline. Recognising this, Bernice Neugarten proposed a more specific classification in the 1970s, dividing older adults into three subgroups: young-old (60–69 years), middle-old (70–79 years), and old-old (80 years or older) [25]. Therefore, the findings of this study are initially applicable to young-old women. To analyse the predictive value of isometric strength for 1RM, a randomised split-sample design was employed. Both groups exhibited similar physical characteristics, minimising potential biases due to intergroup differences and strengthening the validity of our findings.

The women in our study had a high body mass index (BMI), indicating that a significant proportion of participants fell into the overweight or obese category. This finding is consistent with the reality in Mexico, where 70% of adults over 60 years old are classified as overweight or obese [26]. Hence, a significant number of participants were classified as overweight or obese, a condition that could influence their physical performance and affect the relationship between isometric strength and 1RM.

To assess functional capacity and measure the effects of training, especially in older adults, it is essential to know the reliability of the instruments used for this population. The ICC provides an indication of the reliability of the instrument. In our study, young-old women demonstrated an excellent intraclass correlation coefficient (ICC = 0.96) in test repetitions, indicating that this assessment is highly reliable for measuring isometric muscle strength in this population. In the literature, maximum isometric strength assessment in older adults is often conducted using isokinetic devices, which are commonly available in rehabilitation settings. These devices have also shown high reliability in measuring knee extensor strength [27,28,29,30]. MVIC assessment is a simple and practical alternative, as it can be performed with more accessible and cost-effective devices compared to isokinetic equipment. These instruments use a load cell (strain gauge) placed between the tested limb and the evaluator’s hand, allowing the participant to exert pressure without generating movement. Despite their simpler design, these devices have also demonstrated excellent repeatability and intra-session reliability in strength measurements [19,20,31,32].

When interpreting the magnitude of changes between measurements taken at different time points, and considering instrument accuracy, the SEM and MDC prove to be more precise indicators than the ICC. For this reason, it is common to find studies reporting these metrics in functional tests applied to older adults, both healthy and those with medical conditions [33,34,35,36]. The SEM measures the random error of the method, meaning variations that cannot be attributed to either the evaluators or the participants. On the other hand, the MDC represents the threshold beyond which the difference between two measurements can be considered real with a given level of confidence, typically 95% [37]. In our study, when assessing MVIC in leg extension, we found that the inherent error of the method was approximately 4.3%. Additionally, for a difference between successive measurements to be considered real and not attributable to instrument error, it must be at least 11.9%. These values are comparable to those previously reported in both young adults [38,39] and older adults [20,40].

The 1RM assessment is widely regarded as the gold standard for evaluating and prescribing strength training [41]. However, its application in older women may face several challenges, one of which is the need to lift submaximal and maximal loads. This demand can create a sense of insecurity and reduce the willingness of the participants to complete the test. Additionally, caregivers and healthcare professionals often cite multiple barriers to its implementation. These include time constraints, lack of proper equipment, absence of trained personnel, high workload, limited experience with the protocol, insufficient resources, and the overall complexity of the procedure. These factors can hinder the use of 1RM testing in this population, despite its value as a tool for both assessment and strength training planning [42], or raise concerns about safety, particularly regarding acute cardiovascular complications [43,44]. Given these limitations associated with 1RM assessments, alternative methods have been sought to provide safer and more practical means of evaluating muscle strength.

MVIC assessment offers several advantages by eliminating the need to handle external loads or execute high-intensity dynamic movements. Additionally, MVIC has been shown to have a high correlation with 1RM across various populations, including young adults (r = 0.78) [45], recreational athletes (r = 0.97) [46], collegiate American football players (r = 0.61–0.72) [47], and patients with multiple sclerosis (r = 0.897) [23]. In our study (Table 3), we found a strong correlation (r = 0.786) between maximum voluntary isometric contraction (MVIC) and one-repetition maximum (1RM) in the study group, suggesting that isometric strength could serve as a predictor of 1RM. The coefficient of determination (R^2^ = 0.618) indicates that 61.8% of the variability in 1RM can be explained by isometric strength, while the remaining 38.2% may be influenced by other individual factors not measured in this study. The 1RM prediction of the model deviates from the actual values by ±6.9 kg in the study group. When validating the model (1RM = 0.932 MVIC − 3.852) in the test group (Table 4), no significant differences were found between the estimated and measured 1RM (*p* = 0.791). This suggests that the equation developed in this study could be applicable to populations with similar characteristics. However, beyond the *p*-value, it is important to note that the average prediction error for 1RM in this group was approximately 13.4%, similar to the error observed in the study group. While this predictive model may be considered useful, the degree of imprecision in the prediction should not be overlooked.

This study provides fitness and strength training professionals working with older women with both the minimal detectable change (MDC) value for isometric strength measurement in knee extensors and a potentially useful model for predicting 1RM. However, there are some limitations, including the sample composition, which consisted exclusively of women aged 60 to 69 years, limiting the generalizability of the results to older populations. In addition, the load displacement during the 1RM assessment was not monitored using a linear position transducer but was instead determined through the supervision of an experienced evaluator, and a considerable proportion of participants were classified as overweight or obese. Future studies should compare MVIC repeatability and its predictive value for 1RM in both men and older women, as well as incorporate additional variables, such as prior strength training experience, to improve prediction accuracy. Additionally, further research should examine whether the relationship between isometric strength and 1RM remains stable or changes following strength training programs in older women.

## 5. Conclusions

The results of this study indicate that measuring the maximum isometric strength of the knee extensors using a load cell is highly reliable in young-old women, supporting the use of this methodology for assessing this aspect of strength in this population. Additionally, isometric strength shows a strong correlation with dynamic maximum strength (1RM), suggesting that isometric strength could serve as a valid predictor of 1RM, facilitating the assessment of this capacity. However, predicting 1RM from MVIC involves a degree of imprecision that must be considered in practical applications. Future studies are needed to refine the predictive model by incorporating additional factors not addressed in this research.

## Figures and Tables

**Table 1:** Physical characteristics of the participants, with the sample divided into the study and validation groups.

Variable	All (*n* = 82)					Study Group (*n* = 41)					Validation Group (*n* = 41)					*p*	*d*	
	Mean	±	SD	Min	Max	Mean	±	SD	Min	Max	Mean	±	SD	Min	Max			
Age (years)	66.9	±	4.1	60.0	75.0	66.9	±	4.2	60.0	75.0	67.0	±	4.0	60.0	74.0	0.98		0.01
Weight (kg)	71.2	±	11.4	49.5	96.6	71.0	±	11.5	49.5	96.3	71.3	±	11.3	52.8	96.6	0.92		0.02
Height (m)	1.6	±	0.1	1.4	1.8	1.57	±	0.06	1.43	1.70	1.56	±	0.05	1.45	1.75	0.78		0.06
BMI (kg/m^2^)	29.0	±	4.7	20.6	42.4	28.8	±	4.6	20.6	41.7	29.2	±	4.8	21.9	42.4	0.73		0.08
MVIC (kg)	49.9	±	10.5	31.4	72.5	48.2	±	9.3	31.4	66.1	51.5	±	11.5	34.5	72.5	0.16		0.31
1RM (kg)	42.5	±	11.5	20.0	70.0	41.1	±	10.9	21.0	60.0	43.9	±	12.0	20.0	70.0	0.28		0.24

BMI: body mass index; MVIC: maximum voluntary isometric contraction; 1RM: One Repetition Maximum; SD: standard deviation; Min: lowest values; Max: highest values; *p*: *p*-value; *d*: Cohen’s *d* value.

**Table 2:** Intra-session reliability of isometric strength measurements.

	MVIC	Mean (kg)	SD	ICC	Confidence Interval 95%		CV (%)	SEM (kg)		SEM (%)	MDC (kg)	MDC (%)
All	Test 1	48.6	10.7	0.960	(0.937	0.974)	3.2		2.1	4.3	5.8	11.9
	Test 2	49.3	10.4									

MVIC: maximal voluntary isometric contraction; SD: standard deviation; ICC: intraclass correlation coefficient; CV: coefficient of variation; SEM: standard error of measurement; MDC: minimal change detectable.

**Table 3:** Linear regression equation for the study group.

	Slope	Intercept (Beta)	R	R^2^	SEE (kg)
Study group	0.932	−3.852	0.786	0.618	6.9

R: correlation coefficient; R^2^: coefficient of determination; SEE: standard error of estimate.

**Table 4:** Comparison of measured and estimated 1RM values in the study and validation groups.

Groups	Measured_1RM (kg)			Estimated_1RM (kg)			*p*-_1_	△ (%)			*p*-_2_
Study group	41.1	±	10.9	41.1	±	8.6	0.996	14.6	±	17.2	0.758
Validation group	43.9	±	12.0	44.2	±	10.7	0.791	13.4	±	17.3	

1RM: One Repetition Maximum (kg); Estimated 1RM (Kg): estimated using the regression equation; *p*-_1_: *p*-value between measured 1RM and estimated 1RM; Δ: Absolute percentage error between 1RM measured and estimated; *p*-_2_: *p*-value comparing the absolute percentage errors between group and validation groups.

## Data Availability

The datasets used and/or analysed during the current study are available from the senior author on reasonable request, japazf@unileon.es.

## Abbreviations

MVIC	Maximum Voluntary Isometric Contraction
1RM	One-Repetition Maximum
ICC	Intraclass Correlation Coefficient
SEM	Standard Error of Measurement
MDC	Minimal Detectable Change
R^2^	Coefficient of Determination
*p*	*p*-value
SG	Study Group
VG	Validation Group
BMI	Body Mass Index
Min	Minimum
Max	Maximum
CV	Coefficient of Variation
SEE	Standard Error of Estimate
Δ	Absolute Percentage Error
d	Cohen’s d
